# Identification of Breed Differences in Known and New Fescue Toxicosis Associated Phenotypes in Charolais-and Hereford-Sired Crossbred Beef Cows

**DOI:** 10.3390/ani11102830

**Published:** 2021-09-28

**Authors:** Kayla M. Lucas, Dawn A. Koltes, Laura R. Meyer, John D. Tucker, Donald S. Hubbell, Jeremy G. Powell, Jason K. Apple, James E. Koltes

**Affiliations:** 1 Department of Animal Science, Iowa State University, Ames, IA 50010, USA; kaylucas@iastate.edu (K.M.L.); delkins@iastate.edu (D.A.K.); 2 Department of Animal Science, University of Arkansas, Fayetteville, AR 72701, USA; lauraruthmeyer@gmail.com (L.R.M.); johndtucker005@gmail.com (J.D.T.); dhubbell@uark.edu (D.S.H.III); jerpow@uark.edu (J.G.P.); jason.apple@tamuk.edu (J.K.A.); 3 Division of Agriculture Livestock and Forestry Research Stations, University of Arkansas, Batesville, AR 72501, USA; 4 Department of Animal Science and Veterinary Technology, Texas A&M University-Kingsville, Kingsville, TX 78363, USA

**Keywords:** beef cattle, fescue toxicosis, genetic robustness, hair shedding, tall fescue, vaginal temperatures

## Abstract

**Simple Summary:**

The consumption of toxic fescue has caused significant losses in the U.S. beef industry. Widely accepted symptoms of toxic fescue exposure include the retention of a thick hair coat, tissue necrosis in the extremities, and reduced nutrient absorption. However, there is variability in the severity of these symptoms, both across and within breeds. The objective of this study was to characterize the effect of fescue toxicosis across Hereford- and Charolais-sired cows for known and new fescue stress-associated phenotypes. Results indicated that Hereford cows had a lessened ability to shed their winter coat and regulate body temperature along with lower serum mineral concentrations compared to Charolais cows when exposed to toxic fescue. Differences between and within sire breed in hair shedding were also observed, providing further evidence of genetic variation. This study provides evidence of variability in the fescue toxicosis that is potentially useful for genetic selection to reduce fescue stress and characterizes effects on pregnant cows which may impact calves in utero.

**Abstract:**

Beef cattle phenotypes are affected by the consumption of toxic fescue. Toxic fescue’s impact is dependent on heat stress and breed composition, with genetic variability for robustness to toxin exposure believed to exist within and across breeds. The study objective was to characterize the effect of fescue toxicosis across breeds for known and novel heat and fescue stress-associated phenotypes. One-hundred crossbred fall-calving Charolais- and Hereford-sired cows of parities 1–3 were allocated to graze either toxic fescue (*n* = 50), non-toxic fescue (*n* = 25), or a rotation between toxic and non-toxic fescue (*n* = 25) for 156 days. Phenotypes impacted by breed (genetics) included hair coat score (*p* < 0.0001), hair reduction/shedding rate (*p* < 0.05), rectal temperature (RT) (*p* < 0.0001), vaginal temperature (*p* < 0.05), serum phosphorus concentration (*p* < 0.02) and respiration rate (RR) (*p* < 0.003). Cows on toxic fescue experienced reduced hair shedding efficacy (*p* < 0.0001), higher vaginal temperatures (*p* < 0.0001), increased systolic blood pressure (*p* < 0.04), increased RR (*p* < 0.0001) and reduced average daily gain (*p* < 0.0001), compared to cows grazing non-toxic fescue. Calves born from cows with higher RT during the last third of gestation had higher RT at weaning (*p* < 0.02), indicating potential physiological effects of in utero heat stress. The study indicates that beef cows exhibit variable responses to toxic fescue within and across breeds which may impact future calf phenotypes.

## 1. Introduction

Tall fescue (*Lolium arundinaceum* aka *Schedonorus arundinaceus*) is a staple forage in southeastern U.S., feeding over 8.5 million cattle [1]. Toxic fescue’s heartiness against drought and heavy grazing is largely attributable to a fungal endophyte, *Epichloë coenophiala* [2]. The fungus produces ergot alkaloids which interact with biogenic amine receptors, inducing a variety of adverse physiological effects in grazing livestock [3,4], collectively referred to as fescue toxicosis [5]. Recognized indicators of fescue toxicosis include the retention of a thick hair coat, suppressed serum prolactin, vasoconstriction in the extremities (tissue necrosis), and reduced feed intake and nutrient absorption [5,6]. These symptoms work in combination with heat stress to suppress growth, conception rates, milk yield, and inhibit thermoregulation [7,8]. While these indicator phenotypes provide insight into the underlying physiology of fescue toxicosis, variation exists between individuals exposed to similar conditions.

Attempts to mitigate fescue losses have involved limiting exposure to toxic pastures by replanting low toxin cultivars or removing cattle from toxic pastures [9,10]. Replanting fescue is costly and time consuming; thus, the selective breeding of cattle for robust performance on toxic fescue would improve the utilization of existing pastures. Genetic variation in thermoregulation and robustness traits on toxic fescue has been documented between *Bos taurus* and *Bos indicus* cattle. *Bos indicus* cattle have shown resistance to both heat and fescue toxin challenge [11], along with variation among sire lines within breeds for tolerance to ergot alkaloids [12,13,14,15,16]. However, current measures of fescue toxicosis symptoms lack resolution to capture underlying genetic variation for fescue toxicosis tolerance.

Several documented indicators of fescue toxicosis are either confounded with heat stress, subjective in nature, lack reliable estimates of genetic variation, or are not practically implemented by producers. Livestock reactions to ergot alkaloid exposure are largely dependent on ambient temperatures [17]. Fescue toxicosis phenotypes which are often confounded with heat stress include increased rectal temperatures, respiration rates and reduced average daily gain. Notable symptoms to differentiate between fescue toxicosis and heat stress include hair coat score and vasoconstriction in the extremities. However, hair coat scores are subjective in nature, as they rely on a single point-in-time measurement by an evaluator. Suppressed serum prolactin, which has been linked to reduced hair shedding ability and pregnancy status [18,19,20,21,22], also lacks reliable estimates of genetic variation. The development of fescue foot or tissue necrosis in the extremities is a notable indicator of fescue toxicosis; however, it lacks a reliable estimate of genetic variation. Doppler ultrasound blood flow measurements have been suggested as a means of quantifying vasoconstriction in the caudal vein, but this technology is too costly and only addresses one of the many physiological effects of fescue toxicosis; thus, it is not practically implemented by producers [23]. While these traits provide insight into the underlying physiology of fescue toxicosis, variation exists between individuals, and even sire lines, exposed to similar conditions. This variation contributes greatly to our lack of understanding about fescue toxicosis. Therefore, the development of new, reliable phenotypes is needed to: (1) better understand the underlying physiology of fescue toxicosis, (2) capture the underlying genetic contributions to fescue toxicosis susceptibility and tolerance, and (3) identify all cattle experiencing fescue toxicosis and not simply those with extreme phenotypes.

In addition to the identification of new traits affecting the individual consuming toxic fescue, it is imperative that the impacts of in utero exposure on calf health are also addressed. Beef producers have long stated that animals not acclimated to toxic fescue fail to thrive and perform poorly when first introduced to the forage. In dairy cattle, in utero heat stress is known to impact life-long animal health, growth, culling rates and productive longevity [24,25]. Since toxic fescue exacerbates the impact of heat stress, it is likely that calves from beef cows experience some physiological effects of heat stress in utero, but the effects on calves are unknown to date. The objectives of this study were: (1) to determine if Charolais- and Hereford-sired crossbred cows differ for documented indicators of heat stress and fescue toxicosis, (2) identify the impact of toxic fescue on new traits, and (3) investigate the effect of toxic fescue exposure in utero on calf performance.

## 2. Materials and Methods

Cows in this study were reared at the University of Arkansas Division of Agriculture Livestock and Forestry Research Station in Batesville, Arkansas and handled in accordance with the regulations of the University of Arkansas Institute for Animal Care and Use Committee under protocol number 16037.

### 2.1. Animals and Experimental Design

A total of 100 crossbred fall-calving cows were included in a 156-day (March to August) grazing trial in 2016. Cows were distributed to graze toxic fescue pasture (>300 parts per billion (ppb) ergovaline), novel fescue (non-toxic) or alternating (rotating from toxic fescue to novel fescue weekly) for the duration of the trial. The alternating pasture was referred to as TNT for the toxic–novel–toxic rotation. Each pasture was subdivided into two 12- to 13-acre paddocks, resulting in a stocking rate of 0.5 acre/cow with 4 toxic fescue paddocks, 2 novel paddocks and 2 TNT paddocks. One paddock in each pasture pair contained a pond, resulting in pond access every other week for each cow, as the pasture management included weekly rotations. Pastures were tested for ergovaline by the University of Missouri Veterinary Diagnostic Laboratory (Columbia, MO, USA.) Cows were grouped by breed (*n* = 58 and 42 for Charolais- and Hereford-sired cows, respectively) and parity [(1st (*n* = 54), 2nd (*n* = 21), or 3rd (*n* = 25)] across the three pasture types. Cow parity was based on a cow’s parity number the previous calving season, as cows were culled from the herd when confirmed open. Prior to the initiation of the trial, cows grazed on non-toxic pastures for at least four months and were artificially inseminated with subsequent natural service cover in December 2015. Multiple (i.e., >5 sires) artificial insemination and natural service sires were used for breeding per breed. A total of 81 cows were confirmed pregnant and open cows remained on the grazing trial. All cows were subjected to heat stress (particularly in July and August), as observed from ambient temperatures recorded for a large portion of the trial (>72 temperature-humidity index [26,27]) (Supplemental File S1).

Two major sampling and phenotyping events were performed on day 0 (March; pre-exposure and low ambient temperatures) and day 156 (August; post-exposure and elevated ambient temperatures). Measurements collected pre- and post-exposure included rectal temperature (RT), respiration rate (RR), body weight (BW), body condition score (BCS), and hair coat score (HCS). Post-exposure only phenotypes collected included blood pressure and blood serum mineral concentrations. Phenotypes recorded by the same two observers at least once monthly on days 0, 28, 57, 83, 112, 140, 149, and 156 included HCS and BCS. Serial vaginal temperature collection was conducted between days 149 and 156 of the trial.

### 2.2. Phenotyping Techniques

Blood pressure was collected using an Omoron digital wrist blood pressure monitor with wrist cuff (Omron Healthcare Co.; Muko, Kyoto, JP, USA). The wrist cuff was secured around the coccygeal blood vessels [28] until a reading was obtained. Blood was collected into a BD Vacutainer with NH trace elements sodium heparin (Greiner Bio-One North America Inc., Monroe, NC, USA) tube from the jugular vein. Serum was separated and serum minerals were analyzed by Fayetteville Agriculture Diagnostic Laboratory in Fayetteville, AR. BW were collected using a TRU-Test model 702 monitors with MP series 600 load bars (Datamars, Lamone-Lugano, Ticino). The BW collected on day 0 and day 156 were used to calculate average daily gain (ADG). RR (breaths/minute) were calculated based on breath counts in a 15s interval multiplied by four. BCS (scale of 1–9 with 1 = emaciated and 9 = obese [29]) were estimated by two observers at least once monthly. HCS were estimated as described by [30] [scale of 1–5 with 1 = slick summer coat (100% shed) and 5 = full, thick winter coat (0% shed)] by two observers. The *HCS* observations throughout the trial were adjusted using a sliding window average to capture underling trends in hair shedding to reduce evaluator and date of observation bias. A sliding average is calculated such that each adjusted *HCS* value is equal to the average of the original observation, the original observation preceding, and the original observation following. The rate of hair reduction (*HRR*) over the trial was estimated using the adjusted *HCS* from days 0 and 156 (Equation (1)).

(1) $$HRR=\left(\frac{adjusted\;HCS\;on\;Day\;156\;–adjusted\;HCS\;on\;Day\;0}{156\;days}\right)$$

RT were collected using a GLA M700 Digital Thermometer, Ser.# M3198 (GLA Agricultural Electronics, San Luis Obispo, CA, USA). Cow RT were recorded in March and August of 2016 and calf RT were taken at weaning in April 2017. Serial vaginal temperatures were collected using iButton DS1922L thermosensors (Digi-Key, Thief River Falls, MN, USA) embedded in progesterone-free controlled internal drug release devices (CIDR) (Zoetis, Parsippany-Troy, NJ, USA), similar to methods presented by [31]. The temperature collection protocol was programed using the OneWireViewer program (Maxim Integrated, San Jose, CA, USA). Thermosensors were labeled with an animal identification code, synchronized to a computer clock, and programed to collect temperatures every five minutes at a resolution of ± 0.5 °C. Sensors were secured in the CIDR by slicing the silicone casing at the center groove and embedding the sensor within. The sensor was secured with electrical tape and groves were sealed with a waterproof silicone sealant (LOCTITE 2.7-oz Specialty Adhesive). CIDR were placed intravaginally on day 149 and removed on day 156. Vaginal temperatures from six days were used to remove the effects of handling stress and summary statistics of serial temperatures were analyzed, including average, variance, maximum, minimum, and daily area under the temperature curve (AUC). Daily vaginal temperature AUC estimates were calculated using a trapezoidal method, where the rectangular base represented the change in time, in hours, and the height was the difference in maximum and minimum vaginal temperatures; the resulting statistic is reported in degrees Celsius per hour °C*h and represents an individual’s accumulated “heat load” over time. Five daily AUC measures were averaged to calculate an individual cow’s average AUC measurement per day.

### 2.3. Statistical Analysis

All models were fit using PROC GLIMMIX in SAS 9.4 (SAS Institute, Carry, NC, USA). The threshold for significance was considered at *p* < 0.05 and tendencies were considered at *p* < 0.10. Pairwise comparisons between estimated least square means between cows from each fescue pasture type were adjusted using Tukey. Models for single record quantitative variables (models (2a) and (2b)) were analyzed using a base model, with fixed effects for fescue pasture exposure type (*Trt*), sire breed (*Breed*), pregnancy status (*Preg*), parity (*Parity*), and an interaction between fescue exposure type and sire breed (*Trt* × *Breed*). The March phenotype (*MarchCovariate*) was included in the model to allow for standardization based on an animal’s phenotype at the start of the trial (i.e., pre-exposure and low ambient temperatures). To further understand the fescue exposure effect on rectal temperature, a fixed effect for pond was nested within fescue exposure type [*Pond* (*Trt*)], as the use of a pond could significantly impact these results:

(2a) $$Y_{ijkl}=\mu +Trt_{i}+Breed_{j}+Preg_{k}+Parity_{l}+Trt_{i}\times \;Breed_{j}+\varepsilon_{ijkl}$$

(2b) $$Y_{ijklm}=\mu +Trt_{i}+March\;Covariate_{m}+Breed_{j}+Preg_{k}+Parity_{l}+Trt_{i}\times Breed_{j}+\varepsilon_{ijklm}$$

Repeated measures for hourly vaginal temperature (model (3)) were analyzed with the base model having the same fixed effects as above and where the cow was fit as a random effect. Six days of temperature data were analyzed to exclude handling bias. In this model, fescue exposure type now considers pond effect and is represented by *TRT*2 and day of serial vaginal temperature recording (*Date*) is included along with relevant interactions between *TRT*2 and *Date* and between *TRT*2, *Date*, and *Breed* in an attempt to capture non-additive variation in vaginal temperatures, due to fescue exposure and pond availability, day of measurement, and sire breed. Serial vaginal temperatures were recorded during times of pond usage, so the *Trt*2 variable creates four exposure categories for the comparison of cow phenotypes: toxic pasture with a pond, novel pasture with a pond, toxic pasture with no pond, and alternating pasture (*TNT*) with no pond. The vaginal temperature model was fit using the BY statement to estimate vaginal temperature for each hour:

(3) $$Y_{ijklm\;}=\mu +Trt2_{i}+Breed_{j}+Preg_{k}+Parity_{l}+Date_{m}+Trt2_{i}\times Breed_{j}+Trt2_{i}\times Breed_{j}\times Date_{m\;}+\varepsilon_{ijklm}$$

Calves sired by SimAngus and Brangus bulls were used to analyze the effect of maternal heat stress in utero. Crossbred cows grazing toxic (*n* = 32), novel (*n* = 23), and *TNT* (*n* = 14) fescue pastures were compared for the effect of toxic fescue exposure and heat stress on maternal RT. Data were analyzed where calf body temperature at weaning was tested for effects of fescue exposure type (*Trt*), RT of the dam 1–2 months prior to calving (*DamRT*), dam sire breed (DSB), parity of dam (*Parity*), and calf sire breed (*CSB*). This model allowed the impact of toxic fescue and sire breed differences to be accounted for to focus on the impact of maternal heat stress (model (4)).

(4) $$Y_{ijklm}=\mu +Trt_{i}+DamRT_{j}+DSB_{k}+Parity_{l}+CSB_{m}+\varepsilon_{ijklm}$$

## 3. Results

### 3.1. Analysis of Known Phenotypes in Response to Fescue Exposure

#### 3.1.1. Average Daily Gain and Body Conditioning

A summary of the differences in phenotypes observed between cows grazing on toxic fescue, novel fescue, or TNT pastures is provided in Table 1. The interaction between sire breed and fescue exposure was not significant for ADG (*p* > 0.05). As expected, ADG was altered by exposure to toxic fescue (*p* < 0.001), where cows on novel fescue gained approximately 0.24 kg/day more (*p* < 0.0001) than their counterparts on toxic fescue. Similarly, cows on TNT pastures gained 0.27 kg/day more than cows grazing toxic pastures (*p* < 0.0001). There was no difference observed between cows on novel fescue and the TNT group for ADG (*p* > 0.05).

August BCS of cows was significantly altered by exposure to toxic fescue (*p* < 0.001). Cows were found to have a 0.68-point higher BCS when grazing on novel fescue (*p* < 0.0003) and a 0.73-point higher BCS on TNT pasture (*p* < 0.0001) than toxic fescue when March BCS (i.e., initial BCS of cow at trial start) was modeled as a covariate. At pre-exposure sampling (March), Charolais-and Hereford-sired cows had different BCS (*p* < 0.005), with Hereford-sired cows being scored, on average, 0.39 points (*p* < 0.005) higher than the Charolais-sired cows. At post-exposure sampling (August), Charolais-sired cows gained 0.16 kg/day more (*p* < 0.0005) than Hereford-sired cows but had similar BCS (*p* > 0.05) (Table 2). The interaction between sire breed and fescue exposure was not significant for August BCS (*p* > 0.10).

#### 3.1.2. Physiological Measures of Heat Stress/Fescue Toxicosis

Blood pressures were recorded after 5 months of fescue exposure. Systolic blood pressure was altered by toxic fescue exposure (*p* < 0.04), whereas diastolic blood pressure was not (*p* > 0.05). Systolic blood pressure was observed to be 107.13 mm of mercury (mmHg) (*p* < 0.04) in cows which grazed novel fescue versus 130.68 mmHg (*p* < 0.0001) in those on toxic fescue (Table 1). However, there was no significant difference between the two groups for diastolic blood pressure (*p* > 0.1). No breed differences were recorded for either measurement of blood pressure (*p* > 0.10).

August RR was affected by fescue exposure (*p* < 0.0001). RR was 70.56 breaths/min (*p* < 0.0001) for those on novel fescue, 55.60 breaths/min for cows on TNT pasture (*p* < 0.0001), and 81.13 breaths/min (*p* < 0.0001) for cows on toxic fescue. There were significant differences in RR for cows on novel versus TNT pasture (*p* < 0.05) and toxic versus TNT pasture (*p* < 0.0001). At pre-exposure sampling, Charolais- and Hereford-sired cows did not have different RR (*p* > 0.1). Post-exposure, Charolais-sired cows had RR of 62.03 breaths/min (*p* < 0.0001) compared to 76.16 breaths/min for Hereford-sired cows (*p* < 0.0001) (Table 2).

#### 3.1.3. Hair Coat Score

**Figure 1:**
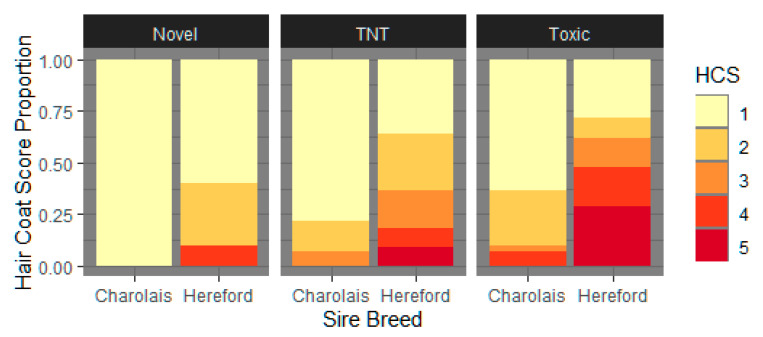
August hair coat scores (HCS) differ by fescue pasture type and sire breed. Cow HCS were evaluated using the scoring system described by [30] [1 = slick summer coat (100% shed) to 5 = full, thick winter coat (0% shed)]. The proportion of August (heat stressed/end of trial) HCS classifications by sire breed (Charolais or Hereford) and fescue exposure type (novel, toxic-novel rotation (TNT), or toxic) were significant (*p* < 0.002 and *p* < 0.0001, respectively) when March (cool weather/beginning of trial) HCS were modeled as covariates to adjust for initial variable in hair.

Least square means for HCS for each sire breed and fescue type for the length of the trial are listed in Table 3. Hair coat scores were similarly pre-exposed (day 0; *p* > 0.10) until day 53 (*p* > 0.10), with differences in HCS seen as early as day 28 (*p* < 0.02) between sire breeds. August HCS were significantly different depending on fescue exposure (*p* < 0.02) and sire breed (*p* < 0.0001) in Figure 1. Charolais-sired cows had, on average, a final HCS of 1.27 ± 0.193 (*p* < 0.0001), while Hereford-sired cows averaged HCS of 2.368 ± 0.225 (*p* < 0.0001). There was no evidence (*p* > 0.10) of an interaction between fescue exposure and sire breed.

#### 3.1.4. Rectal Temperature

There was no difference in March RT (*p* > 0.10), but after 5 months’ exposure to toxic fescue, August RT was significantly altered (*p* < 0.0001). RT was 0.90 °C higher for both groups of cows on novel fescue (*p* < 0.0001) and toxic fescue (*p* < 0.0001) compared to those on the TNT pasture. There was no significant difference in RT between the novel and toxic groups (*p* > 0.1). To validate these findings, a second model was run, this time considering ponds nested within a fescue exposure type. Here, the overall effect of fescue exposure remains the same as specified previously; the novel fescue (*p* < 0.0001) and toxic fescue (*p* < 0.0001) groups were 0.90 °C warmer than those on the alternating pastures, with no difference between the two (*p* > 0.1). At pre-exposure sampling, Charolais- and Hereford-sired cows did not have differences for RT (*p* > 0.1), but post-exposure (Table 2) Charolais-sired cows had 0.58 °C lower RT (*p* < 0.0001).

#### 3.1.5. August HCS as Covariate for Rectal Temperature

**Figure 2:**
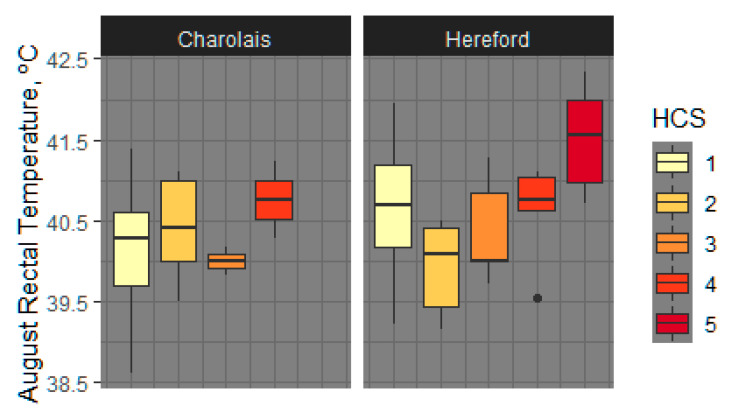
August cow rectal temperatures increase as hair coat score (HCS) increases and differ by sire breed. August HCS (*p* < 0.004) and sire breed (Charolais or Hereford) were found to have significant (*p* < 0.004) effects on August rectal temperature when analyzed independently of fescue exposure type. Effect of August HCS was seen in comparison of rectal temperatures for cows with an August HCS of 5 to those with HCS 1, 2 or 3.

To determine the impact of HCS on RT, it was included as a model covariate to determine if differences in body temperature were impacted by hair shedding. HCS (*p* < 0.004) and sire breed (*p* < 0.04) were found to have significant effects on August RT when analyzed regardless of fescue type (Figure 2). The effect of August HCS on RT was most evident in comparison of cows with HCS 5 to cows with either HCS 1, 2, or 3. On average, cows with HCS 5 had RT 1.10 °C higher than those with HCS 1 or 2 (*p* < 0.003 and *p* < 0.005, respectively) and RT 1.22 °C higher compared to HCS 3 (*p* < 0.009). Hereford-sired cows were found to have 0.35 °C higher (*p* < 0.04) RT than Charolais-sired cows, regardless of fescue exposure, when August HCS was modeled as a covariate.

### 3.2. Analysis of New Phenotypes in Response to Fescue Exposure

#### 3.2.1. Blood Serum Mineral Concentration

For measured serum minerals, only phosphorus (*p* < 0.0001) and magnesium (*p* < 0.0001) were influenced by fescue exposure. Both phosphorus and magnesium serum levels were 0.97 milligram per liter (mg/L) (*p* < 0.0007) and 0.19 mg/L (*p* < 0.003) lower, respectively, in cows which grazed novel fescue than those on the TNT pasture. Additionally, phosphorus levels were 1.74 mg/L higher (*p* < 0.0001), and magnesium levels were 0.21 mg/L higher (*p* < 0.0001) for cows on TNT pasture than toxic fescue. Serum phosphorus levels were 0.77 mg/L higher (*p* < 0.006) for cows on novel pasture when compared to those on toxic pasture, with no difference found for serum magnesium levels (*p* > 0.1) (Table 1). Charolais-sired cows had 0.56 mg/L higher serum phosphorus (*p* < 0.02) concentrations than Hereford-sired cows.

#### 3.2.2. Hair Reduction Rate

**Figure 3:**
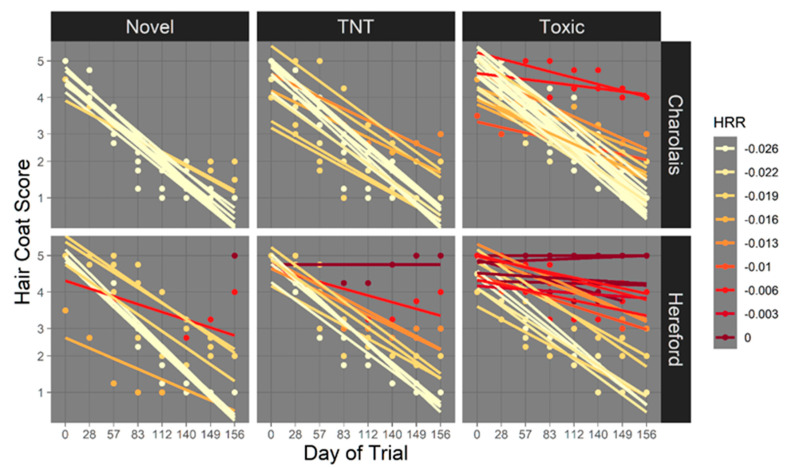
Individual cow variation in hair reduction rate (HRR) by fescue type and sire breed. Cow hair coat score (HCS) was scored monthly using the system described by [30] [1 = slick summer coat (100% shed) to 5 = full, thick winter coat (0% shed)]. HRR was calculated by performing a sliding window average of HCS to get adjusted HCS for each time point. Adjusted HCS from day 0 and day 156 were used to calculate rate of hair shed over time, of HRR. Each line represents the change in HCS over change in time (days) for each cow. The top and bottom panels represent rate of hair reduction for Charolais- and Hereford-sired cows (*p* < 0.0001) on novel fescue, toxic-novel rotation (TNT), and toxic fescue, respectively (*p* < 0.0004). Variability on novel fescue within the Herefords may indicate across-family variability in hair shedding. Variability in shedding in both breeds on TNT and toxic pastures may indicate genetic differences across families when exposed to fescue mycotoxins.

After HCS were adjusted via a sliding window average, the HRR were calculated resulting in 9 unique rates of hair reduction over the course of the trial, with individual variation in HRR by sire breed (*p* < 0.0001) and fescue type (*p* < 0.0003) represented in Figure 3. At post-exposure, HRR was slowed for Hereford-sired cows (−0.016 HCS/day, *p* < 0.0001) compared to Charolais-sired cows (−0.023 HCS/day, *p* < 0.0001). Least square means for variation in hair reduction efficacy for fescue type can be found in Table 1 and Table 2 for fescue exposure and sire breed, respectively. Overall, cows on toxic fescue had reduced shedding ability of −0.016 HCS/day (*p* < 0.0001). There was no interaction between sire breed and fescue type for HRR (*p* > 0.1).

#### 3.2.3. Serial Vaginal Temperature

**Figure 4:**
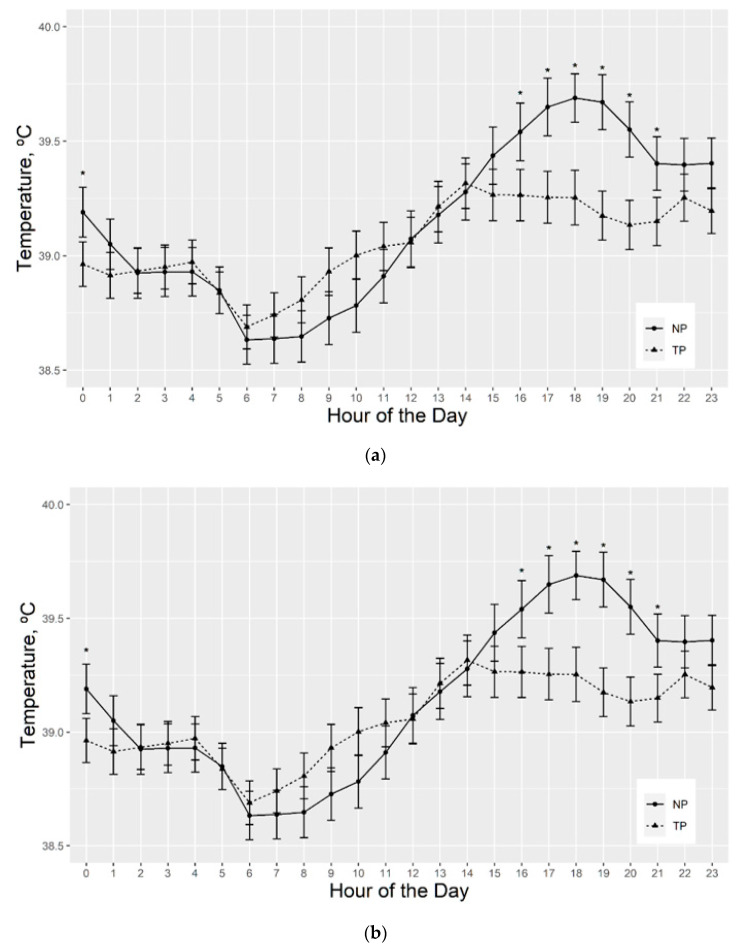
(**a**). Least square means for repeated measures of vaginal temperature of cows grazing toxic fescue pasture with a pond (TP) compared to cows on toxic fescue pasture without a pond (TNP). The last week of the trial, after cows had grazed either toxic or novel fescue for 5 months, cow vaginal temperatures were recorded using iButton thermosensors (Digi-Key, Thief River Falls, MN, USA) in progesterone-free Controlled Internal Drug Releasing Device (CIDR) (Zoetis Parsippany-Troy, NJ, USA). Temperatures were captured every 5 min at a sensitivity of ± 0.5 °C. Six days of temperatures were analyzed to exclude handling bias. Tendencies for significant pond effect on vaginal temperature, by hour of the day, at *p* ≤ 0.06 are indicated by an asterisk above the error bar. (**b**). Least square means for repeated measures of vaginal temperature of cows grazing toxic fescue pasture with a pond (TP) compared to cows on novel fescue pasture with a pond (NP). The last week of the trial, after cows had grazed either toxic or novel fescue for 5 months, cow vaginal temperatures were recorded using iButton thermosensors (Digi-Key, Thief River Falls, MN, USA) in progesterone-free Controlled Internal Drug Releasing Device (CIDR) (Zoetis Parsippany-Troy, NJ, USA). Temperatures were captured every 5 min at a sensitivity of ± 0.5 °C. Six days of temperatures were analyzed to exclude handling bias. Significant toxic exposure effect on vaginal temperature, by hour at *p* ≤ 0.05, is indicated by an asterisk over the error bar.

Repeated measures analysis of serial vaginal temperature had a significant (*p* < 0.05) fescue exposure effect from 1600 until 2000 h (data not shown). For this analysis, the following contrasts were considered: (1) cows grazing toxic fescue with and without access to a pond (Figure 4a); (2) cows grazing toxic pasture with a pond compared to those on novel fescue with a pond (Figure 4b); and (3) cows on toxic pasture without access to a pond compared to those on the alternating pasture with no pond access (Supplemental File S2).

For the cows on toxic pasture, those which did not have access to a pond averaged vaginal temperatures 0.15 °C warmer than cows on toxic pasture with access to a pond, with tendencies (*p* < 0.06) for differences being found in vaginal temperature for these groups between 1500 and 2100 h (Figure 4a). The average daily AUC heat accumulation was 4.7 °C*h higher (*p* < 0.04) for the cows on toxic pasture without pond access than those on toxic pasture with pond access.

On a novel pasture with pond access, vaginal temperatures were higher (*p* ≤ 0.05) at 0000 h and 1600 through 2100 h than cows on toxic fescue with pond access (Figure 4b). Temperature differences ranged from 0.23 °C (*p* < 0.06) at 0000 h to 0.50 °C (*p* < 0.0003) at 1900 h. There was no difference (*p* > 0.1) in average daily AUC heat accumulation for these groups. For the last comparison, cows on toxic pasture and TNT pasture without pond, there was no difference found (*p* > 0.05) in vaginal temperature for these groups at any hour of the day (Supplemental File S2) or in average daily AUC heat accumulation (*p* > 0.01).

**Figure 5:**
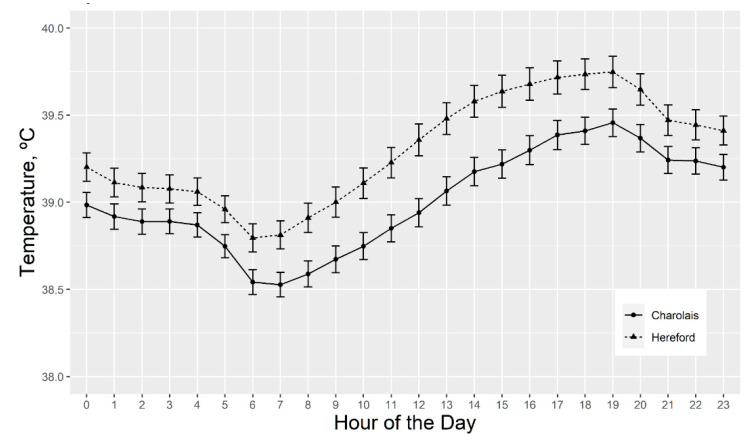
Least square means for repeated measures of vaginal temperatures for Charolais- and Hereford-sired cows, regardless of fescue treatment type. The last week of the trial, after cows had grazed either toxic or novel fescue for 5 months, cow vaginal temperatures were recorded using iButton thermosensors (Digi-Key, Thief River Falls, MN, USA) in progesterone-free Controlled Internal Drug Releasing Device (CIDR) (Zoetis Parsippany-Troy, NJ, USA). Temperatures were captured every 5 min at a sensitivity of ±0.5 °C. Six days of temperatures were analyzed to exclude handling bias. Significant breed effect on vaginal temperature, by hour at *p* ≤ 0.05, is indicated, where error bars do not overlap.

Significant (*p* < 0.05) breed effects for the repeated measures analysis of serial vaginal temperatures are reported in Figure 5. Hereford-sired cows averaged vaginal temperatures 0.30 °C higher (*p* < 0.05) than Charolais-sired cows at all hours of the day. Additionally, Charolais-sired cows had a 0.28 °C lower vaginal temperature mean (*p* < 0.003), a 0.42 °C lower maximum temperature (*p* < 0.0001), a 0.31 °C smaller temperature range (*p* < 0.0006), and a 0.05 °C2 smaller vaginal temperature variance (*p* < 0.003) than Hereford-sired cows. Lastly, the average daily area under the curve (AUC) heat accumulation was 4.13 °C*h higher (*p* < 0.002) for the Hereford-sired cows (Table 2).

#### 3.2.4. August HCS as Covariate of Serial Vaginal Temperature

**Figure 6:**
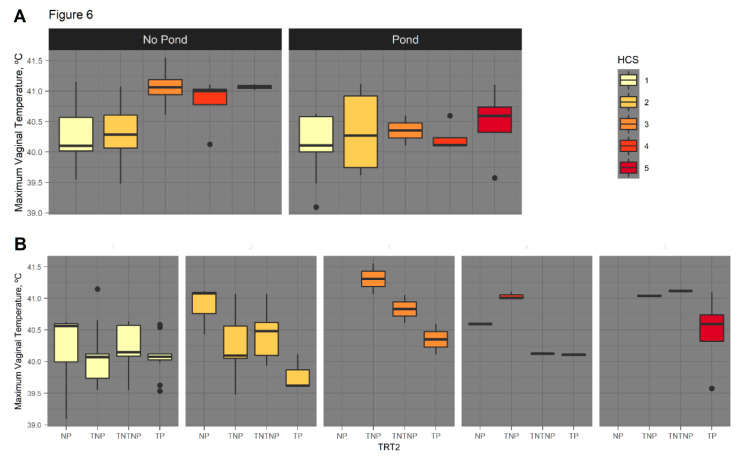
Serial vaginal temperature maximums increase as August hair coat scores (HCS) increase and vary with pond availability and fescue treatment. As serial vaginal temperatures were recorded during times of pond usage, the Trt2 variable creates four treatment categories: toxic pasture with a pond (TP), novel pasture with a pond (NP), toxic pasture with no pond (TNP), and TNT with no pond (TNTNP). (**A**) Cows were grouped solely on pond availability, with HCS 1 through 5 represented in each group. Cows with no pond access indicate a positive correlation between August HCS and maximum vaginal temperature, with a reduced ability to reach thermoneutral, as indicated by the smaller temperature range for all HCS. Cows with pond access have similar maximum temperatures, indicating pond usage as a cooling mechanism, regardless of HCS. (**B**) Maximum vaginal temperatures by August HCS and TRT2 classification. Cows on toxic fescue not only have elevated maximum vaginal temperatures, but are more likely to retain thicker HCS, further exaggerating this difference.

Since HCS was identified as a significant factor impacting RT, models of vaginal temperature differences were developed to account for HCS. When modeled with August HCS as a covariate, there was a tendency (*p* < 0.1) for an interaction between TRT2 and August HCS for vaginal temperature maximums; however, there were no significant differences (*p* > 0.05) in multiple comparisons for the interaction. When the interaction was dropped, cows with HCS of 1 were found to have 0.56 °C lower (*p* < 0.05) maximum vaginal temperatures compared to those with HCS of 3 and 0.59 °C lower than cows with HCS 5 (*p* < 0.04). There was no difference (*p* > 0.1) found for maximum vaginal temperature between cows in the HCS 1 and 4 groups (Figure 6).

Similarly, for vaginal temperature variance measurements, when an interaction between TRT2 and August HCS was modeled, there was a tendency (*p* < 0.1) for an effect. Cows on novel pasture with pond access with HCS 2 tended to have a vaginal temperature variance 0.20 °C^2^ wider (*p* < 0.1) than those on the toxic pasture with pond access with HCS 2. Most interesting was the wider variation of 0.21 °C^2^ in vaginal temperatures for cows on novel pasture with HCS 2, than cows on toxic pasture with HCS of 4 (*p* < 0.04). The impact of HCS as a covariate of serial vaginal temperature means can be found in Supplemental File S3.

### 3.3. Comparison of Trait Correlations between Toxic, Alternating (TNT), and Novel Fescue Pastures

Pearson’s correlations for heat and fescue stress phenotypes, contrasted by fescue pasture, can be found in Table 4. For the cows on toxic fescue, a strong positive correlation existed between HRR and RR (*p* < 0.0001), but the same correlation was not significant for cows on novel fescue (*p* > 0.10). Similarly, cows on toxic fescue had a strong positive correlation between HRR and RT (*p* < 0.0001), but the correlation between HRR and RT was non-significant and negative (*p* > 0.10) in the novel group. Additionally, for cows on toxic fescue, there were no significant correlations (*p* > 0.10) found between RR and any of the serial vaginal temperature statistics reported. However, correlations between RR, and all but one of the serial vaginal temperature statistics, tended to be significant (*p* ≤ 0.10) in the novel group.

Correlation plots contrasted by cows on TNT and novel fescue, and TNT and toxic fescue, indicate that the relationships between heat and fescue stress phenotypes for cows on TNT pasture may more closely resemble those of cows on toxic fescue than novel fescue. Correlation plots between novel fescue and toxic fescue pastures, TNT and toxic pastures, and TNT and novel pastures can be found in Supplemental Files S4–S6, respectively.

### 3.4. Impact of Cow Body Temperature on Calf Body Temperature Later in Life

**Figure 7:**
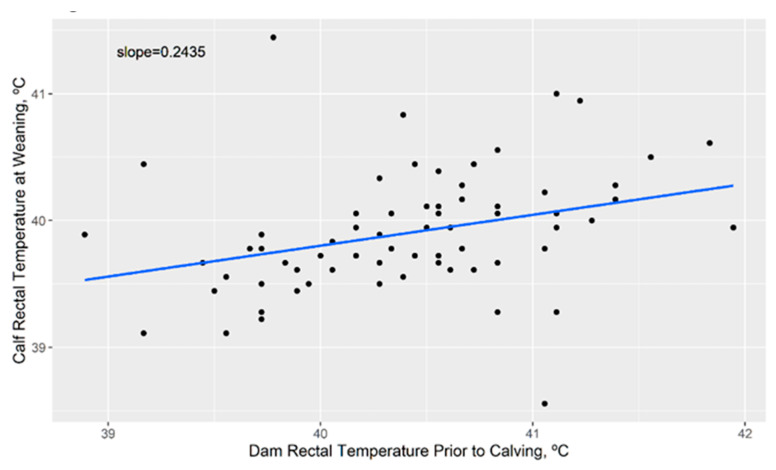
Calf rectal temperature at weaning is significantly associated (*p* < 0.05) with dam rectal temperature prior to calving, regardless of novel or toxic fescue exposure. Cow rectal temperatures were taken 1–2 months prior to calving. These data may indicate the genetic or epigenetic tuning of calf temperature in response to heat stress in utero.

Investigation into the effect of maternal RT during the third trimester of gestation on calf RT at weaning showed that calves born from cows with higher body temperatures had significantly higher body temperatures at weaning (*p* < 0.02) (Figure 7). There was a significant exposure effect (*p* < 0.04) where calves from cows grazing toxic fescue had RT 0.33 °C higher at weaning than those on novel fescue (*p* < 0.03). To examine the true effect of maternal RT, a statistical model was run with data only from animals on novel fescue. This analysis showed that the effect of maternal RT remained significant (*p* < 0.04) regardless of toxin exposure on calf RT at weaning.

## 4. Discussion

Today, toxic fescue can be found throughout the continental U.S. [32]. As it spreads and average ambient temperatures continue to rise, more severe symptoms of fescue toxicosis in U.S. beef cattle are expected. For these reasons, and based on the findings of this study, we believe that more whole-animal phenotyping is needed in animals on toxic fescue, to better characterize the impact of fescue toxicosis and identify those traits which are most useful for selecting robust cattle. The impact of toxic fescue on animal physiology is complex, with a variable expression of phenotypes impacted, even within a breed. Both Charolais and Hereford breed groups are impacted by toxic fescue; however, the magnitude of effect tended to be lower in Charolais cattle. Results illustrated variation within breed groups, indicating potential family differences within breeds. Along with breed, effects of toxic fescue exposure have been identified, both for phenotypes known to be associated with fescue toxicosis (e.g., ADG and HCS) and novel phenotypes (e.g., serum blood minerals and vaginal temperature) not previously associated with toxic fescue exposure. Increases in body temperature associated with toxic fescue have been identified, but were more impacted by HCS than toxic fescue exposure. Interestingly, correlations between traits associated with toxic fescue exposure differ across pasture types. This may indicate fescue exposure’s impact on animal physiology changes how one trait impacts another trait. In addition to these findings this study is among the first to identify a long-lasting effect on calves exposed in utero to heat stress, with high RT at weaning in calves born from cows with higher RT in their 3rd trimester of pregnancy. These results were exacerbated by toxic fescue exposure.

### 4.1. Weight Gain and Body Condition Traits Differed by Toxic Fescue Exposure and Sire Breed

Loss in growth efficiency is one of the major economic impacts of fescue toxicosis. In this study, we evaluated weight gain during pregnancy while under heat stress (>72 temperature-humidity index [26,27]), under three fescue pasture conditions. Although, [21] found no difference in feed intake between steers fed toxic and novel fescue, [33,34] found that animals on toxic fescue spend more time standing, loafing or seeking cooling, leading to lower feed intake, especially under heat stress. Our findings were consistent with the latter, suggesting that cows on either novel fescue or TNT were more likely to graze, even under heat stress, allowing them to maintain a higher ADG and subsequent higher BCS than cows on toxic fescue. Further research is needed to determine the effect of reduced body weight gain and maintenance in cows, while grazing toxic fescue on their gestating calves’ performance.

### 4.2. Respiration Rates Were Impacted by Fescue Type and Sire Breed and Blood Pressure Were Impacted by Toxic Fescue

Respiration rates were highest for cows on novel pasture, while cows on TNT had the lowest RR. Additionally, Hereford-sired cows had higher RR than Charolais-sired cows. These findings differ from a previous study, which found RR to be increased in cattle consuming toxic fescue over novel fescue [22]. These results could be due to cows on novel pasture spending less time seeking cooling and more time grazing throughout the day, meaning that they rely on panting and/or sweating as a means of heat transfer from the body [35]. However, more research on behavioral patterns while grazing toxic fescue are needed to differentiate if increased RR in cows on novel fescue relative to those on toxic fescue are due to increased activity levels (e.g., more time grazing) or to lower body temperature during periods of heat stress due to not utilizing the available pond as frequently.

The impact of toxic fescue on blood pressure is different than heat stress, since fescue increases vasoconstriction [23,35], while heat stress increases vasodilation [36]. Thus, blood pressure is among the few phenotypes associated with fescue toxicosis and not heat stress. Contraction of the vascular system is thought to be caused by the interaction of ergot alkaloids with serotonin receptors in smooth muscle, leading to implications for heat dissipation, blood pressure, and heart rate [5,35]. An increase in systolic blood pressure, as seen in cows on toxic fescue versus those on novel, can lead to other health issues in cattle, such as congestive heart failure [37].

### 4.3. Blood Serum Phosphorus and Magnesium Are Impacted by Sire Breed and Toxic Fescue Exposure

Hypophosphatemia (20–40 mg/L phosphorus) and hypomagnesemia (11–18 mg/L magnesium) have been recorded in cattle with depressed feed intake [37,38,39] and under heat stress [40,41]. In the present study, cows on all fescue types had serum phosphorus and magnesium levels below normal levels, indicating heat stress [40]. A difference between novel and toxic groups existed for serum phosphorus levels and Charolais-sired cows had higher phosphorus levels than Hereford-sired cows, with no difference in serum magnesium levels. Hypocalcemia has been reported in conjunction with hypomagnesemia and hypophosphatemia in cattle experiencing heat stress [41]. Although serum calcium levels were not significantly effected in this study, there is evidence that the downregulation of prolactin from the ingestion of toxic fescue [6] restricts calcium uptake [42], to which magnesium concentration is correlated [43,44]. The relationship between prolactin, magnesium, calcium, and other regulatory elements brings to question the effect toxic fescue has on mineral metabolism and homeostasis in grazing beef cattle. Alternatively, the differences seen in serum mineral concentrations pre- and post-exposure could be due to an electrolyte imbalance as a results of heat stress.

It has been documented that sheep and steers fed high-endophyte diets had reduced blood flow to absorptive surfaces of the rumen compared to those fed low-endophyte diets [35,40], leading to decreased nutrient uptake, as indicated by reduced ADG for cows grazing toxic fescue compared to those on the TNT pasture, which was accompanied by decreased serum phosphorus and magnesium absorption [45,46,47]. Furthermore, cows on novel fescue had decreased phosphorus and magnesium levels compared to those on TNT pastures, which is inconsistent with previous publications. This observation is thought to be linked to differing grazing behaviors between the groups; however, as grazing behavior was not monitored in the present study, this theory cannot be corroborated. Unlike serum phosphorus concentration, a reduction in magnesium concentration was only associated with a decreased ADG in cows on toxic fescue.

### 4.4. Hair shedding and HCS Are Impacted by Toxic Fescue Exposure and Exhibit within And across Sire Breed Variability

Thick summer hair coats on toxic fescue have been determined to be comprised of new hair growth and reduced shedding [48]. Reduction in hair shedding ability during the summer months is among the hallmarks of fescue toxicosis [5,6]. Our findings are consistent with these publications, as cows grazing toxic fescue pastures had thicker HCS than those grazing either TNT or novel fescue pastures [22,48,49]. Additionally, Hereford-sired cows have August HCS higher than Charolais-sired cows on all fescue pasture types. Potential bias in our findings for coat color between sire breeds was not found to be a factor in our study, as variation in both HCS and HRR within both sire breeds were evident. Charolais-sired cows also experienced higher rectal and vaginal temperatures with increased HCS, suggesting that HCS is a driving factor in core body temperature, regardless of fescue type or genetic influence.

To reduce HCS sensitivity to date and evaluator bias intrinsic to hair coat scoring, adjusted hair coat scores and then HRR (i.e., a slope of hair shedding over time) were calculated for each cow. This adjustment provided higher resolution for capturing individual variation in hair shedding capability. Fescue exposure differences existed, such that cows on novel fescue exhibited the most efficient HRR (i.e., fastest shedding rate), cows on toxic fescue showed the least efficient HRR (i.e., slowest shedding rate), and cows on TNT pastures were intermediate, consistent with the limited ergot alkaloid exposure of this group.

### 4.5. Rectal and Vaginal Temperatures Differ by Sire Breed, Hair Coat Thickness, and Tend to Differ by Fescue Pasture Type

Increased RT is one possible indicator of heat stress, which can occur at lower ambient temperatures in animals grazing toxic fescue than those grazing non-toxic forage [17,50]. In March, RT were similar for all cows regardless of sire breed, pregnancy status and parity. August RT were higher in Hereford-sired cows than Charolais-sired cows and, most surprisingly, cows on novel fescue had similar August RT to cows on toxic fescue. This discrepancy could be due to low power in our statistical model for the detection of a difference between cows on novel and toxic fescue, as the sample size for the novel fescue group was half of that for toxic fescue. These results can further be explained by the relationships between fescue exposure and HCS [22,48,49] and sire breed and HCS [30]. As previously discussed, fescue exposure type influenced HCS and HRR such that cows on toxic fescue have higher August RT, which were positively correlated with a higher HCS. Meanwhile, cows on novel fescue had lower HCS and may not have felt the effect of high ambient temperatures, leading them to continue grazing, and accumulate higher RT as a result of standing in direct sunlight for a greater number of hours in a day [50]. Presumably due to differences in hair coat color, sire breed effects were observed, where the Hereford-sired (dark coat) cows had higher August RT than the Charolais-sired (light coat) cows [50,51]. Measurements for RT can also be influenced by environmental instability, as bringing in grazing beef cows increases body temperature beyond what is typical, due to handling stress. Thus, body temperatures were also evaluated with vaginal sensors to eliminate the impact of human interaction with animals on body temperature.

Limitations exist in the interpretation of serial vaginal temperatures in this study, due to the inclusion of the TNT pasture resulting in the uneven allocation of pond by fescue exposure for the period where vaginal temperatures were recorded. However, pond effects on toxic and novel fescue pastures could be evaluated. Vaginal temperatures were hypothesized to be influenced by sire breed, HCS, pasture type and pond access. Vaginal temperatures are known to be influenced by hair coat, along with temperament and weight [52], further supporting our findings. Wetting of the hair coat and skin surface as a means to quickly reduce core body temperatures [53] was hypothesized to be observed within our study, as cows with pond access were consistently cooler during the hottest time of day between 1500 (−0.25 °C) and 1800 (−0.41 °C) hours, while fescue exposure was significant, but varied throughout the day.

Vaginal temperatures were evaluated to identify differences in temperature in response to fescue pasture exposure; however, differences identified may indicate differences in grazing behavior [54]. In our results, cows with access to a pond, no matter the fescue exposure type, had reductions in vaginal temperatures during the afternoon, presumably due to utilization of the pond. This is especially evident between those on novel fescue with pond compared to the toxic fescue with pond. As the toxic group cool off, the novel group continues to rise in temperature, indicating that they likely spent more time grazing, or at least potentially grazing. Corroborating this hypothesis, the animals on novel pasture with pond access and TNT animals without pond have increased ADG. On the contrary, cows on toxic fescue had an increase in vaginal temperatures in the mid-morning hours with a steady increase as the day progresses. This may suggest that those on the toxic fescue spend the cooler hours of the morning grazing and thus, experience a rise in core body temperature as heat production from microbial fermentation accounts for 3 to 8% of total heat production in cattle [55], leading cows to seek cooling throughout the hotter parts of the day. Activity data could further confirm results presented in this study, as an animal’s activity level could impact multiple phenotypes measured, including RR, RT, and vaginal temperature. This provides justification for future studies, which may aim to monitor both grazing behavior and activity level of cattle consuming toxic fescue pastures to aide in the identification of animals experiencing symptoms of fescue toxicosis.

Heat stress has a major impact on animal fertility [56]. It has been reported that cows on toxic fescue have a conception rate of 55% compared to 96% for cows grazed on endophyte-free pastures [57]. Proposed explanations for failure to conceive include lowered serum progesterone, poor blood flow, and compounded effects of heat stress and fescue toxicosis, including increased core body temperatures [5,12]. Results from this study indicate that animals on toxic fescue tend to be hotter due to higher HCS, with cows that have not shed their winter coat (HCS 5) having maximum vaginal temperatures 0.41 °C to 0.60 °C higher than cows with lower hair scores. Such large differences in body temperature are very likely to negatively impact cow reproductive performance, as conception rates dropped from 52% (38.2 °C) to 12% (40.0 °C) [58].

### 4.6. Calf Body Temperature at Weaning Is Impacted by Maternal Heat Stress

Fall calving programs have been suggested to reduce economic losses from reduced calf growth traits on toxic fescue [59]. Unfortunately, calves are then exposed to in utero heat stress from their dams during summer gestation. Holstein calves which have been exposed to in utero heat stress exhibited not only higher core body temperatures, such as the calves did in our study, but also weaker immune systems and lower levels of fertility later in life [25]. This further exacerbates economic losses on toxic fescue due to higher levels of culling, due to illness and/or inability to conceive. Further studies are needed to determine the impact of in utero heat stress in beef cattle, which may differ due to different selection and physiology in beef versus dairy breeds. However, the results of this study are a baseline for determining the impacts of the dam’s fescue exposure on calf productivity.

### 4.7. Evidence of Genetic Differences in Hereford-and Charolais-Sired Cows in Response to Toxic Fescue

Results of this study suggest that Hereford cattle are more severely impacted by toxic fescue. This was indicated by phenotypes recorded which have previously been associated with both fescue toxicosis and heat stress such as higher HCS through the summer months, increased RR and RT, and decreased ADG. Additionally, this study serves to highlight genetic differences for blood serum mineral concentrations and serial vaginal temperatures.

There is strong evidence that genetics impact hair shedding. Brahman-influenced cattle had faster hair shed than cattle of European descent, with estimates of heritability of hair shed to be 0.35 [30] and 0.34 to 0.40 for cattle on toxic fescue [46]. Sire breed effects may have been identified in this study, as Hereford-sired cows had a reduced efficacy in hair shedding than Charolais-sired cows on all fescue pasture types, with this difference being exaggerated on toxic fescue. Interestingly, there appears to be clustered patterns in the slope of HRR for cows within both breeds. This is most evident within Hereford-sired cows, as there appear to be three distinct groupings of HRR for Hereford-sired cows on toxic fescue. The grouping of HRR and differences among the Hereford group may indicate that different cows with the same sire may have similar HRR, and thus similar susceptibility to ergot alkaloids, with variability in hair shedding being a possible function of sire.

A study by [60] compared progeny of a Hereford sire, reputed to have progeny resistant to ergot alkaloid exposure, to another sire with unknown records. Each group of calves were fed endophyte-infected tall fescue, and their physiological responses were recorded, including their ability to maintain and transfer body temperature. There were no differences found between the two, but calves sired by the bull with a reputation for toxin resistance showed greater feed intake and lower RT throughout the trial. This outcome led the authors to conclude that the overall performance of these calves caused the reputation of the sire’s offspring to have less susceptibility to fescue toxicosis. Similarly, our results indicate that Charolais-bred cattle may be genetically robust to toxic fescue as indicated by higher ADG, lower HCS and subsequent core body temperatures than Hereford-sired cattle.

Interactions between fescue exposure and sire breed were not significant for any of the phenotypes recorded. There were tendencies for a sire breed by fescue exposure interaction for August HCS, which can be seen in comparisons of Charolais- and Hereford-sired cows on all fescue pasture types, with Hereford-sired cows having higher HCS and more linear HRR. The presence of toxins makes the genetic differences more evident between and within the breeds. Other tendencies are observed between August HCS and fescue TRT2 for serial vaginal temperature maximums and variances. As HCS increases, vaginal temperature maximums increase, which can also be related to the sire breed effect, as we have seen evidence of genetic influence on HCS and HRR. With this, serial vaginal temperature variances are wider for cows on novel pasture with pond access than cows on toxic pasture with pond access with identical HCS, providing evidence for a sire breed effect in the ability to maintain thermoregulation through hair shedding ability.

## 5. Conclusions

The impact of toxic fescue on cows is important to their health and productivity as well as their calves. In this study, level of toxic fescue exposure significantly impacted HCS, HRR, ADG, blood serum minerals (phosphorus and magnesium), vaginal temperature traits and blood pressure. Sire breed differences were identified for HCS, HRR, RR, ADG, BCS, RT, serum blood phosphorus levels and vaginal temperature. There was a tendency towards a breed by fescue exposure interaction for HCS and vaginal temperature when adjusted for HCS. Vaginal temperature measurements of cows on toxic pasture with a pond indicate that cows on toxic pasture spend more time in the pond during the hottest part of the day which may limit grazing; however, additional studies are needed to verify effects of pond by pasture type on grazing behavior. Our results between and within sire breeds indicate that genetic variation exists for selection to reduce the impact of heat and fescue stress, especially for HCS and HRR. The variability in phenotypes impacted by fescue toxicosis underscores the need for additional studies to characterize the whole-body effects of ergovaline and explore the impact of toxic fescue beyond HCS. Furthermore, the impact of cow temperature on calf body temperature later in life, regardless of fescue toxin exposure, indicates that additional studies are needed to determine how heat and fescue stress may impact the long-term health and productivity of cows and calves.

## Figures and Tables

**Table 1:** Least square means ± standard error for phenotypes significantly impacted by fescue pasture type.

	Novel	TNT ^1^	Toxic	*p*-Value *
N	25	25	50	
**August single time point phenotypes**				
Systolic Blood Pressure, mmHg	107.1 ± 9.412 ^a^	119.6 ± 8.036 ^ab^	130.7 ± 6.253 ^b^	0.0383
Rectal Temperature, °C	40.64 ± 0.159 ^a^	39.73 ± 0.130 ^b^	40.62 ± 0.109 ^a^	<0.0001
Respiration Rate, breaths/min	70.56 ± 5.203 ^a^	55.60 ± 4.253 ^b^	81.13 ± 3.573 ^a^	<0.0001
Serum Phosphorus Concentration, mg/L ^2^	6.775 ± 0.249 ^a^	7.744 ± 0.203 ^b^	6.005 ± 0.171 ^c^	<0.0001
Serum Magnesium Concentration, mg/L ^2^	2.233 ± 0.048 ^a^	2.422 ± 0.039 ^b^	2.203 ± 0.033^a^	<0.0001
**Serial Phenotypes over time**				
Average Daily Gain, kg/day	0.692 ± 0.050 ^a^	0.720 ± 0.041 ^a^	0.452 ± 0.034 ^b^	<0.0001
Hair Coat Score ^3^	1.356 ± 0.286 ^a^	1.748 ± 0.231 ^ab^	2.352 ± 0.199 ^b^	0.0011
Hair Reduction Rate, ∆HCS/day	−0.028 ± 0.002 ^a^	−0.024 ± 0.002 ^a^	−0.016 ± 0.001 ^b^	<0.0001
Body Condition Score ^4^	6.371 ± 0.167^a^	6.418 ± 0.136 ^a^	5.699 ± 0.114 ^b^	<0.0001

^a–c^ Within a row, values without common superscripts denote Tukey-adjusted differences in means (*p* < 0.05). ^1^ Represents the group of cows which alternated grazing novel and toxic fescue. ^2^ Blood samples for serum mineral analysis were collected from the jugular vein on day 156 of the trial, transported on ice, and analyzed at the veterinary diagnostic laboratory (University of Arkansas, Fayetteville, AR). ^3^ 1 = slick, summer coat (100% shed) to 5 = full, thick winter coat (0% shed) [30]. ^4^ 1 = emaciated to 9 = extremely obese [29]. * Overall fescue pasture exposure effect *p*-value. Bold: grouping of phenotypes.

**Table 2:** Least square means ± standard error for significant phenotypic differences in response to sire breed are presented along with pairwise comparisons between sire breeds for each phenotype.

	Sire Breed				
	Charolais	Hereford	Diff ^1^	SE ^2^	*p*-Value ^3^
n	58	42			
**August Phenotypes**					
Average Daily Gain, kg/day	0.701 ± 0.033	0.542 ± 0.0392	0.1586	0.0428	0.0004
Rectal Temperature, °C	40.04 ± 0.107	40.62 ± 0.126	−0.5771	0.1375	<0.0001
Respiration Rate, breaths/min	62.03 ± 3.502	76.16 ± 4.106	−14.125	4.493	0.0023
**Vaginal Temperature, °C**					
Mean	39.02 ± 0.072	39.30 ± 0.082	−0.2793	0.0873	0.0020
Maximum	40.02 ± 0.083	40.43 ± 0.093	−0.4148	0.0997	<0.0001
Range	1.978 ± 0.071	2.286 ± 0.080	−0.3076	0.0852	0.0005
Variance	0.183 ± 0.013	0.233 ± 0.015	−0.050	0.0158	0.0022
Mean daily AUC, °C × h ^4^	23.54 ± 1.052	27.67 ± 1.186	−4.128	1.2669	0.0016

^1^ Pairwise estimate for Charolais–Hereford. ^2^ Standard error for the difference in least square means. ^3^ *p*-value corresponding to the Charolais–Hereford estimate. ^4^ Daily accumulation of degree area under the curve × hours (daily AUC, °C × h). Bold: grouping of phenotypes.

**Table 3:** Fescue type and sire breed effects for hair coat score (HCS) ^1^. (A) Least square means ± standard error for HCS by date of measurement by fescue exposure. (**B**) Least square means ± standard error for HCS by date of measurement by sire breed.

A.	Fescue Type			
	Novel	TNT ^2^	Toxic	*p*-Value
*n*	25	25	50	
Trial Day				
0	4.908 ± 0.063	4.893 ± 0.062	4.817 ± 0.044	0.4045
28	4.400 ± 0.102	4.431 ± 0.101	4.391 ± 0.072	0.9495
57	3.433 ± 0.145	3.621 ± 0.143	3.810 ± 0.102	0.1016
83	2.389 ± 0.168	2.780 ± 0.166	3.361 ± 0.118	<0.0002
112	1.756 ± 0.161	2.264 ± 0.167	3.087 ± 0.119	<0.0001
140	1.511 ± 0.180	2.024 ± 0.178	2.775 ± 0.126	<0.0001
149	1.394 ± 0.194	1.863 ± 0.192	2.443 ± 0.136	<0.0001
156	1.505 ± 0.219	1.815 ± 0.223	2.259 ± 0.161	0.0197
**B.**	Sire Breed			
	**Charolais**	**Hereford**	***p*** **-Value**	
**n**	**58**	**42**		
Trial Day				
0	4.843 ± 0.043	4.902 ± 0.050	0.369	
28	4.269 ± 0.069	4.565 ± 0.082	0.0116	
57	3.340 ± 0.098	3.903 ± 0.116	0.0004	
83	2.505 ± 0.114	3.182 ± 0.134	0.0002	
112	2.037 ± 0.115	2.701 ± 0.135	0.0003	
140	1.756 ± 0.122	2.451 ± 0.144	0.0004	
149	1.456 ± 0.132	2.344 ± 0.155	<0.0001	
156	1.345 ± 0.153	2.394 ± 0.177	<0.0001	

^1^ 1 = slick, summer coat (100% shed) to 5 = full, thick winter coat (0% shed) [30]. ^2^ Represents the group of cows which altered grazing novel and toxic fescue.

**Table 4:** Pearson’s correlation among heat stress and fescue toxicosis phenotypes contrasted by fescue treatment ^1,2^.

	Toxic	TNT	Novel	
	*n* = 50	*n* = 25	n = 25	LEGEND:
**HCS x VTmean**	0.38 *	0.66 *	**0.36**	Positive correlation ≥ 0.50 ^1^
**HCS × ADG**	−0.41 *	−0.61 *	NS	Positive correlation < 0.50 ^1^
**HCS × RR**	0.50 *	NS	NS	Negative correlation ≥ 0.50 ^1^
**HRR × RR**	0.52 *	NS	NS	Negative correlation < 0.50 ^1^
**HRR × RT**	0.50 *	0.52 *	NS	NS (non-significant correlation)
**HRR × VTmean**	0.38 *	0.66 *	**0.38**	HCS (hair coat score, 1 = 100% shed to 5 = 0% shed [30])
**RR × AUC**	NS	NS	0.55 *	VTmean (serial vaginal temperature mean, °C)
**RR × P**	−0.59 *	NS	NS	ADG (average daily grain, kg/day)
**RT × Mg**	NS	−0.53 *	NS	RR (respiration rate, breaths/minute)
**RT × AUC**	NS	0.64 *	**0.39**	HRR (hair reduction rate, ∆HCS/day)
**RT × RR**	0.53 *	NS	0.49*	RT (rectal temperature, °C)
**RT × VTmax**	**0.26**	0.58 *	NS	AUC (average daily accumulation of degree area under the curve × hours, °C × h)
**RT × VTmean**	**0.25**	0.57 *	NS	P (blood serum phosphorus, mg/L)
**RT × VTrange**	NS	0.52 *	NS	Mg (blood serum magnesium, mg/L)
**RT × VTvar**	NS	0.55 **	NS	VTmax (serial vaginal temperature maximum, °C)
**VTmax × Cu**	−0.53 *	NS	NS	VTrange (serial vaginal temperature range, °C)
**VTmax × Mg**	**−0.26**	−0.53 *	NS	VTvar (serial vaginal temperature variance, °C^2^)
**VTmax × Zn**	NS	−0.53 *	NS	Cu (blood serum copper, mg/L)
**VTmean × Zn**	**−0.26**	−0.59 *	NS	Zn (blood serum zinc, mg/L)
**VTmin × Zn**	−0.29*	−0.64 *	NS	VTmin (serial vaginal temperature minimum, °C)
**VTrange × Mg**	NS	−0.50 *	**−0.37**	Na (blood serum sodium, mg/L)
**Na × K**	−0.60 *	NS	−0.41 *	K (blood serum potassium, mg/L)
**Ca × Fe**	0.38 *	0.65 *	0.52 *	Ca (blood serum calcium, mg/L)
**Ca × Zn**	**0.26**	0.52 *	NS	Fe (blood serum iron, mg/L)

^1^ all correlations show are *p* ≤ 0.10 with significant correlations indicated at * *p* ≤ 0.05. ^2^ the coloring with the table differentiates highly positively correlated (red; r > 0.5), positively correlated (pink; 0.25 < r < 0.50) from negatively correlated (light green; −0.5 < r < −0.25) from highly negatively correlated (dark green; r > −0.5). Bold indicate statistical significance.

## Data Availability

The full data presented in this study are owned by the University of Arkansas Division of Agriculture and are available on request from the corresponding author. A summary of all data analyzed are available within the manuscript.

## Supplementary Materials

The following are openly available in the digital repository FigShare. File S1: Daily ambient temperature highs and lows over the course of the 156-day trail, https://doi.org/10.6084/m9.figshare.14787741; File S2: Least square means for repeated measures of vaginal temperatures for cows on toxic pasture without a pond (TNP) compared to cows on an alternating pasture without a pond (TNTNP), https://doi.org/10.6084/m9.figshare.14787750; File S3: As August hair coat score (HCS) increases, serial vaginal temperatures means increase with a pond effect evident, https://doi.org/10.6084/m9.figshare.14787753; File S4: Correlations between novel fescue and toxic fescue pastures, https://doi.org/10.6084/m9.figshare.14787759; File S5: Correlations between TNT and toxic fescue pastures, https://doi.org/10.6084/m9.figshare.14787765; File S6: Correlations between TNT and novel pastures, https://doi.org/10.6084/m9.figshare.14787771.
